# The physiological resilience of fern sporophytes and gametophytes: advances in water relations offer new insights into an old lineage

**DOI:** 10.3389/fpls.2013.00285

**Published:** 2013-08-05

**Authors:** Jarmila Pittermann, Craig Brodersen, James E. Watkins

**Affiliations:** ^1^Department of Ecology and Evolutionary Biology, University of CaliforniaSanta Cruz, CA, USA; ^2^Horticultural Sciences Department, Citrus Research and Education Centre, University of FloridaLake Alfred, FL, USA; ^3^Department of Biology, Colgate UniversityHamilton, NY, USA

**Keywords:** xylem transport, cavitation, desiccation tolerance, ferns, gametophytes

## Abstract

Ferns are some of the oldest vascular plants in existence and they are the second most diverse lineage of tracheophytes next to angiosperms. Recent efforts to understand fern success have focused on the physiological capacity and stress tolerance of both the sporophyte and the gametophyte generations. In this review, we examine these insights through the lens of plant water relations, focusing primarily on the form and function of xylem tissue in the sporophyte, as well as the tolerance to and recovery from drought and desiccation stress in both stages of the fern life cycle. The absence of secondary xylem in ferns is compensated by selection for efficient primary xylem composed of large, closely arranged tracheids with permeable pit membranes. Protection from drought-induced hydraulic failure appears to arise from a combination of pit membrane traits and the arrangement of vascular bundles. Features such as tracheid-based xylem and variously sized megaphylls are shared between ferns and more derived lineages, and offer an opportunity to compare convergent and divergent hydraulic strategies critical to the success of xylem-bearing plants. Fern gametophytes show a high degree of desiccation tolerance but new evidence shows that morphological attributes in the gametophytes may facilitate water retention, though little work has addressed the ecological significance of this variation. We conclude with an emergent hypothesis that selection acted on the physiology of both the sporophyte and gametophyte generations in a synchronous manner that is consistent with selection for drought tolerance in the epiphytic niche, and the increasingly diverse habitats of the mid to late Cenozoic.

## Introduction

Ferns are generally perceived as small-statured plants relegated to the forest understory due to limitations arising from a two-stage life cycle, but in fact, the morphology, habit and life-history of seedless vascular plants (SVPs) varies tremendously with respect to leaf shape, overall stature and leaf longevity (Ranker and Haufler, 2008; Mehltreter et al., 2010). With over 12,000 species of ferns, lycopods and selaginellales in existence, SVPs inhabit desert, tropical, terrestrial, temperate and epiphytic niches where they often effectively compete for resources with conifers and angiosperms (Page, 2002; Coomes et al., 2005; Moran, 2008; Ranker and Haufler, 2008; Chapman, 2009; Mehltreter et al., 2010). It is often overlooked that Pteridophytes (ferns) encompass at least 10,000 species making them the most diverse lineage next to angiosperms. In contrast to their bryophyte ancestors, the evolution of true vascular tissue allowed ferns and other SVPs to display a diversity of leaf shapes and sizes that include arborescent and viney ferns, but it is generally agreed that the period between the Late-Devonian and Carboniferous witnessed the peak of SVP diversity with the evolution of the now-extinct pro-gymnosperms and seed ferns (Taylor et al., 2009; Wilson and Knoll, 2010). Efficient water transport was paramount to the successful colonization of land by plants and one implication is that the appearance of primary xylem in early-diverging SVPs was a key innovation that may have bridged the transition from simple non-vascular plants to the derived woody flora that soon followed (Kenrick and Crane, 1997; Sperry, 2003; Taylor et al., 2009; Pittermann, 2010). Alternatively, recent fossil evidence indicates that the evolution of secondary xylem occurred as early as the Devonian (Gerrienne et al., 2011), so today's SVPs may have capitalized on an early-derived, though successful vascular strategy. Recent analyses suggest that despite their ancient origins, Pteridophytes experienced at least two major post-Cretaceous diversification events that have shaped their biogeography, and potentially their physiology (Schneider et al., 2004; Schuettpelz and Pryer, 2009; Watkins et al., 2010). Many factors have contributed to the continued success of Pteridophytes, but here we explore the water relations of sporophytes and gametophytes, focusing on the structure and function of the xylem tissue and drought-response patterns of the gametophyte stage.

## Water transport in the fern sporophyte

**Figure 1:**
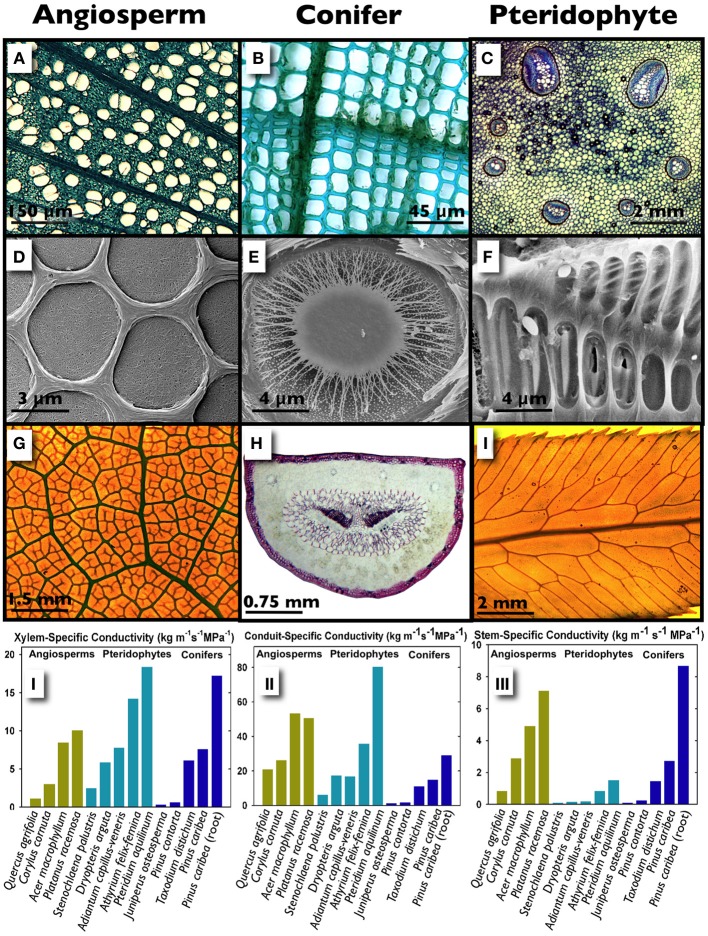
**A structural and hydraulic comparison of angiosperm and conifer stems, and a Pteridophyte stipe.** Transverse sections of diffuse-porous wood in stems of *Platanus racemosa* showing vessels embedded in a dense matrix of fibers (**A**; image courtesy of Prentice), the tracheid-based xylem of *Pinus contorta* **(B)** and the primary vascular tissue arranged in seven bundles in the stipe of *Polypodium aureum*; within the bundles, clear xylem conduits are surrounded by blue-staining phloem (**C**, image courtesy of Rico). The homogoneous pit membranes of *Cladrastris sinensis* (**D**, image courtesy of Jansen), the torus-margo pit membrane of *Sequoia sempervirens* (**E**, image courtesy of Choat) and the scalariform, homogenous pit membranes of *Blechnum brasiliense* (**F**, image courtesy of Carlquist). Leaf venation patterns in *Mahonia aquifolium* **(G)**, *Pinus attenuata* where the two centrally located dark tissues are xylem **(H)**, and the pinnae of *Woodwardia fimbriata* (**I**; images **G** and **I** courtesy of Baer). The graphs illustrate specific conductivities in conifers, angiosperms and ferns whereby conductivity is standardized by the stained (functional) xylem area following a dye perfusion (*Ks;* **I**), the conduit lumen area only **(II)** and the stem/stipe cross-sectional area **(III)**.

Developmentally, fern fronds are megaphylls arising from a rhizome, although in tree ferns the fronds emerge from an apical region atop a trunk comprised of pith parenchyma, fibers and adventitious roots. Fern vascular tissue is packaged in discrete bundles (meristeles) that are surrounded by an endodermis (Figures 1C, 3) and although vessels have been reported in *Pteridium aquilinum* and members of *Astrolepis, Marsilea*, and *Woodsia* (Carlquist and Schneider, 2007; Pittermann et al., 2011), the majority of ferns transport water by means of ancestral tracheids the walls of which are perforated by reticulate, homogenous pit membranes (Figure 1F). The organization of the vascular bundles within the stipe is highly variable, ranging from the solitary vascular central bundle in a haplostele to the multiple bundles that often form a ring in the dictyosteles of some species (Figures 1C, 3). Fern dictyosteles are most commonly reticulate networks (i.e., the bundles are interconnected) with few to potentially many bundles per frond (White and Turner, 1995). In contrast to the typically short and narrow conifer tracheids, which evolved to transport water as well as support the canopy, fern xylem evolved solely for the movement of water leaving support to an outer ring of schlerenchyma fibers. Relieved from a mechanical function, fern tracheids may reach diameters in excess of 100 μm, which is well in the range of angiosperm vessels (Veres, 1990; Pittermann et al., 2006, 2011; Sperry et al., 2006; Watkins et al., 2010). Similarly, the length of fern tracheids varies greatly from 1 mm to 1.5 cm, with conduits in excess of 4 cm observed in scrambling and weedy species (Veres, 1990; Pittermann et al., 2011). The ability of fern xylem to explore a broader morphospace within the constraints imposed by unicellular conduits probably shaped the competitive ability and persistence of the modern pteridoflora, and may have factored into the evolution of pseudo-woody vascular strategies characteristic of extinct Carboniferous fern taxa (Wilson and Knoll, 2010). That said, the absence of a bifacial vascular cambium and its derivative secondary xylem has led to a developmental scheme that limits not only the hydraulic capacity of pteridophytes, but also branching and the overall architecture of the fern canopy.

Recent work suggests that water transport efficiency in ferns can overlap with woody plants, although the majority of ferns fail to reach the hydraulic maxima exhibited by lianas and ring-porous angiosperms (McCulloh et al., 2010; Watkins et al., 2010; Pittermann et al., 2011; Brodersen et al., 2012; Feild and Wilson, 2012). Watkins et al. (2010) surveyed the hydraulic performance of 21 species of tropical ferns occupying both understory and open habitats and discovered that the stipes of terrestrial species exhibit higher rates of xylem area-specific conductivity (*K*_*s*_) than those occupying an epiphytic or an hemi-epiphytic niche, with *K*_*s*_ values ranging from 0.5 to 7 kg m^−1^ MPa^−1^ s^−1^. Surprisingly, there was no relationship between *K*_*s*_ and tracheid diameter in the epiphytes suggesting that xylem transport may be decoupled from the water relations of these ferns. Instead, epiphytes may depend on stored water (Watkins et al., 2010) or foliar water uptake for hydration (Limm et al., 2009; Limm and Dawson, 2010) with xylem transport playing a secondary role. By contrast, temperate terrestrial species such as the cosmopolitan *Pteridium aquilinum*, the large-statured *Woodwardia fimbriata*, and the viney, indeterminately-growing *Lygodium* sp. can exhibit *K*_*s*_ in excess of 20 kg m^−1^ MPa^−1^ s^−1^ (Pittermann et al., 2011; Brodersen et al., 2012; Figures 1.I–III). This is surprising given that fern xylem is ancestral to the secondary xylem found in conifers and angiosperms. What explains the high transport potential in some ferns?

The hydraulic conductivity of a stem is dictated by the components of its functional xylem network including total xylem area, the diameter and length of the xylem conduits, the density of conduits on a xylem area basis (i.e., “conduit packing”), pit membrane features, and the connectivity of the network as a whole (Choat et al., 2008; McCulloh et al., 2010; Brodersen et al., 2011). Ferns possess limited amounts of primary xylem but may maximize hydraulic conductivity by arranging their large conduits in a manner that resembles the tight tracheid packing of conifers (Savage et al., 2010; Pittermann, unpublished data; Figures 1.I, **A–C**). When cell walls, parenchyma and fibers are ignored and *K*_*s*_ is standardized by conduit lumen area alone, angiosperms and ferns can achieve comparably high transport capacities, with even drought-tolerant (*Quercus agrifolia*) and shade-grown (*Carylus cornuta*) angiosperms exhibiting a six- to ten-fold increase in *K*_*s*_, relative to the three- to four-fold increase in fern *K*_*s*_ (Figure 1.II). A high wall fraction in conifers, and the inclusion of non-conductive but structurally important fibers reduces transport capacity and increases the carbon costs of xylem construction in woody plants in a way that is avoided by the primary xylem in ferns. Furthermore, evidence from scanning electron micrographs as well as empirical data suggests that ferns possess pit membranes that are both permeable and abundant along the tracheid walls. In contrast to the densely woven pit membranes of angiosperms (Figure 1D), fern pit membranes appear diaphanous and more porous (Figure 1F)—traits that may further reduce resistance to water flow relative to the high resistance offered by angiosperm pit membranes (Schulte et al., 1987; Wheeler et al., 2005; Carlquist and Schneider, 2007; Choat et al., 2008; Lens et al., 2011; Pittermann and Brodersen, unpublished data). Altogether, the combination of large conduits, tight packing and porous pit membranes is what may allow some ferns to exhibit xylem hydraulic efficiencies that are on par with higher plants (Figures 1.I,II). While connectivity likely plays a significant role in hydraulic conductivity, particularly when water transport needs to be rerouted to bypass blocked or damaged sections of the network, few studies exist that explicitly test these theories (Gibson et al., 1984; Sack et al., 2008; Brodersen et al., 2012).

Limitations in the primary vascular development of Pteridophytes restrict the absolute amount of water transported to the fern frond, placing a fundamental constraint on frond hydraulic function (Figure 1.III). Fern fronds possess a fixed amount of vascular tissue, which means that the xylem irrigates a proportionally scaled range of frond leaf areas, with little to none of the annual flexibility made possible by secondary xylem (Watkins et al., 2010; Pittermann et al., 2011). This developmental bottleneck may explain not only the tight scaling between frond dimensions and leaf area (Limm and Dawson, 2010; Creese et al., 2011) but also the low vein density of the fern pinnae which has been implicated in reduced rates of stomatal conductance and photosynthesis (Brodribb et al., 2007; Figures 1G–I). Furthermore, fern stomata appear to be less dynamic than the stomata of angiosperms, with the implication that ferns are less water-use efficient than derived plants (McAdam and Brodribb, 2011; Brodribb and McAdam, 2011). Hence, intrinsically low water-use efficiencies combined with xylem hydraulic limitations may explain the tendency of most sporophytes to occupy hydric, often shady niches with low evapotranspirative demand (Page, 2002; Cardelús et al., 2006; Watkins et al., 2010; McAdam and Brodribb, 2011, 2013; Pittermann et al., 2011). However, given the co-ordinated water conservation strategies evident in both the sporophytes and their gametophytes within terrestrial and epiphytic functional groups (Watkins et al., 2007a,b, 2010; Watkins and Cardelús, 2012), it is reasonable to expect that the biogeography and ecology of Pteridophytes squarely rests on the physiological co-ordination of these two lifestages.

## Drought tolerance in the sporophyte

**Figure 2:**
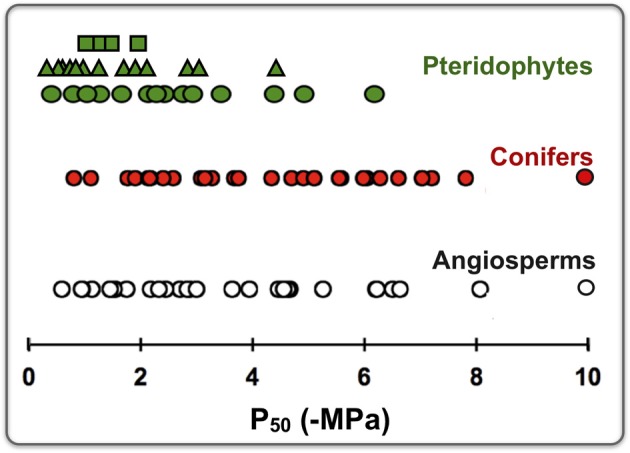
**The water potential causing 50% loss of hydraulic conductivity (*P*_50_) collected from a sampling of conifers, angiosperms and Pteridophytes.** The conifer and angiosperm data were generated using the centrifugal method (Hacke et al., 2001; Pittermann et al., 2006). *P*_50_ values from Pteridophytes were collected using the whole-frond rehydration kinetics method (Brodribb and Holbrook, 2004; squares), air-injection on the stipe (Watkins et al., 2010; triangles) and centrifugation of the stipe (Wheeler et al., 2005; Pittermann et al., 2011; circles).

The degree to which land plants evolved successful strategies for water transport depends in large part on the xylem network's protection from hydraulic failure due to embolism caused by air-seeding. Tension in the xylem sap may exceed some species-specific pressure threshold such that air is sucked through the pit membrane into a water-filled conduit where it expands and subsequently blocks transport (Sperry and Tyree, 1988). Woody plants are variably resistant to embolism depending on their habitat and life-history strategy (Pockman and Sperry, 2000; Pratt et al., 2012) but only a few studies have examined cavitation resistance in ferns. The data indicate that fern xylem is for the most part vulnerable to drought-induced embolism with *P*_50_ values (the water potential at which 50% loss of hydraulic conductivity occurs) generally ranging from −1 to −3 MPa (Figure 2). However, epiphytes and evergreen species adapted to seasonally dry habitats such as the redwood forest understory (e.g., *Polystichum munitum* and *Dryopteris arguta*) may exhibit *P*_50_ values that are substantially lower, indicating a surprising degree of drought resistance that at least in epiphytes, is consistent with measured water potentials *in situ* (Figure 2; Watkins et al., 2010; Pittermann et al., 2011). Gibson et al. (1984) reported an approximately 1 MPa m^−1^ water potential gradient within a single frond, so water potentials *in situ* may vary depending on ambient conditions. Interestingly, data from air-injection and centrifuge-based methods of inducing embolism suggest that fern xylem may possess populations of tracheids that are resistant to air-seeding and potentially act as a back-up transport system during drought stress (Watkins et al., 2010; Pittermann et al., 2011). These tracheids are typically found in the smaller bundles of species such as *D. arguta, P. munitum* and *Woodwardia fimbriata*, but may be present in larger vascular bundles as well (Pittermann et al., 2011; Brodersen et al., 2012; Brodersen and Pittermann, unpublished). It is important to note, however, that most of these data were collected on the basal portion of the frond (the stipe), so workers seeking to understand the ecological significance of embolism resistance in ferns would be wise to evaluate the response of the entire megaphyll, per Brodribb [see Brodribb and Holbrook (2004), McAdam and Brodribb (2013)] or Sack [see Sack and Holbrook (2006), Scoffoni et al. (2011)].

**Figure 3:**
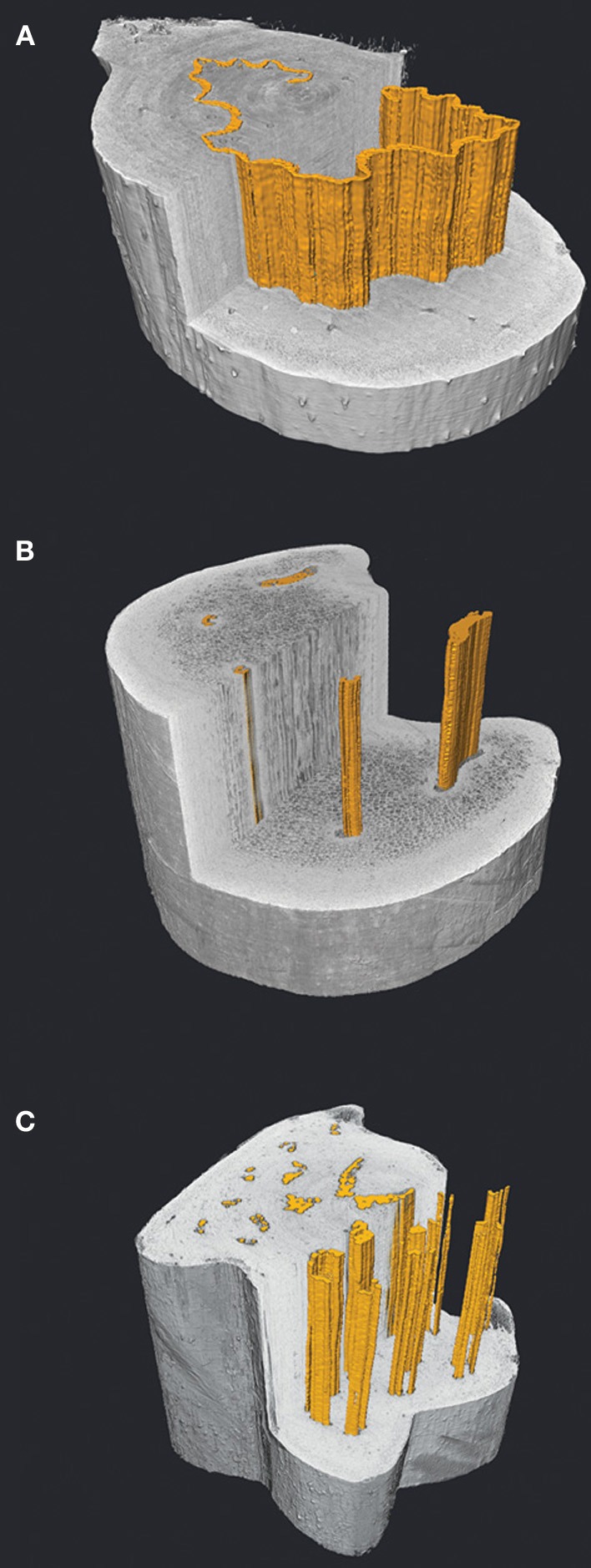
**Virtual transverse and longitudinal sections through frond stipes of *Dicksonia antarctica* (A), *Woodwardia fimbriata* (B) and *Polystichum munitum* (C) visualized with high resolution X-ray computed tomography (HRCT).** The vascular bundles are highlighted in orange and show a small sample of the diversity of stelar patterns in ferns, with the continuous band of xylem in *D. antarctica* **(A)**, to the sectored xylem of *W. fimbriata* **(B)**. Despite the dissected appearance of the xylem in *P. munitum* **(C)**, 3D analysis reveals that the bundles make frequent connections resulting in a highly integrated network (Brodersen et al., 2011). The 3D organization and packing of tracheids along with the xylem associated parenchyma likely play a significant role in determining whether ferns are able to actively restore hydraulic function following xylem embolism.

It is likely that embolism resistance in ferns is governed by degrees of co-variation between xylem arrangement and pit membrane traits. Bundle connections within the frond appear to be functionally comparable to xylem integration patterns in angiosperm trunks, in which greater hydraulic segregation is associated with increasing drought tolerance (Zanne et al., 2006; Schenk et al., 2008). This is because fewer hydraulic connections reduce the possibility of air spreading throughout the xylem network. Brodersen et al. (2012) illustrated this trend in two co-occurring fern species, the drought-deciduous *P. aquilinum* and the perennial *W. fimbriata*, where disparate xylem organization (integrated vs. sectored, respectively) and different life history strategies allow these plants to co-occur in the seasonally low water availability of a coastal California mediterranean climate. Not surprisingly, the seasonally deciduous *P. aquilinum* exhibited higher transport rates, whereas *W. fimbriata* showed greater resistance to embolism (Brodersen et al., 2012). The anatomical differences between these species support these findings. *P. aquilinum* has a complex stele anatomy in which over 20 bundles frequently fuse and bifurcate along the length of the frond (Figure 3C). By contrast, *W. fimbriata* exhibits two large bundles that serve to supply the distal pinnae, with an additional 3–4 smaller bundles forming few if any connections to the large ones (Figure 3B). Instead, these small vascular bundles remain isolated throughout much of the stipe and eventually fuse in the upper portion of the frond, apparently unrelated in function to the pinnae (Gibson et al., 1984; White, 1984; White and Turner, 1995).

While the role of the fern pit membrane in cavitation resistance has not been thoroughly explored, we suspect that the putative fragility of fern pit membranes combined with their abundance along the tracheid walls may render most ferns vulnerable to air-seeding (Figures 1F, 2). In angiosperms, a combination of thin pit membranes and a large pit membrane area increases species' vulnerability to cavitation (Christman et al., 2009; Lens et al., 2011), so given the resemblance in pit membrane structure between angiosperms and ferns, we suspect that the mechanism of cavitation resistance operates under similar constraints in ferns. Interestingly, members of the basal fern genus *Botrychium* exhibit torus-margo pit membranes and developmental patterns that superficially resemble those of conifers (Figure 1E), but the functional significance of these traits is unknown (Morrow and Dute, 1998; Rothwell and Karrfalt, 2008).

An unidentified component to drought and/or desiccation tolerance in ferns is the role of embolism repair. To date, no studies exist that show whether ferns are capable of embolism repair as seen in angiosperms (Zwieniecki and Holbrook, 2009). However, embolism repair appears to be present in angiosperm species with a combination of anatomical and physiological traits that are common in many ferns. Stomatal control and the ability to regulate xylem tension to minimum threshold values close to atmospheric pressure appear to be critical in successful embolism repair in angiosperms (Hacke and Sperry, 2003; Zwieniecki and Holbrook, 2009), as is conduit associated parenchyma to actively refill embolized conduits (Tyree et al., 1999; Brodersen et al., 2011; Nardini et al., 2011). Unlike conifers, fern tracheids are often surrounded by parenchyma tissue and are typically within close proximity to the phloem, thereby increasing the probability of conduit refilling. It may be that the diversity of stelar organization in ferns may favor the refilling process in some species more than others. For example, by orienting phloem tissue adjacently to the xylem, the single thin corrugated vascular bundle in *Dicksonia antarctica* stipes eliminates any space between tracheids and phloem (Figure 3A) as compared to the more derived members of the Polypodiales in which tracheids may be isolated in center of the large bundles (Pittermann et al., 2011; Brodersen et al., 2012; Figures 1C, 3). We suspect that *Dicksonia*'*s* stele arrangement is more amenable to osmotically-mediated embolism repair.

Desiccation tolerant (DT) plants may be ideal candidates for the study of embolism repair in SVPs as well as in higher plants. DT plants can lose over 95% of the water in their tissues, yet completely recover metabolic and photosynthetic activity following exposure to water (Alpert, 2000; Oliver et al., 2000; Proctor and Tuba, 2002). During dehydration, the main axes and the leaves of desiccation tolerant SVPs typically curl and contract, leading to deformation of both parenchyma and possibly the xylem tissue. In the initial stages of rehydration, the re-establishment of positive turgor pressure is coupled to cell membrane, protein and DNA repair, so presumably, the refilling of embolized conduits occurs simultaneously or shortly thereafter. It has been observed that the proto-xylem of mosses (hydroids) does not embolize during dehydration, but rather collapses, similar to the implosion observed in the transfusion tissue of conifer leaves (Proctor and Tuba, 2002; Cochard et al., 2004; Brodribb and Holbrook, 2005). Consequently, the hydroids obviate the need for embolism repair during the re-establishment of hydraulic function. Whether the same can be said of Pteridophytes remains unclear. Pittermann et al. (2011) show that at least in the most hydraulically relevant tracheids, fern xylem does not exhibit structural safety from implosion, while *in situ* observations of xylem function in the fern *Polystichum munitum* reveal no conduit collapse (Brodersen, pers. observation). It appears that cavitation occurs prior to the more negative tensions required for conduit implosion. Some conduit deformation was observed during conduit injection with resin (Pittermann, pers. observation), but whether this occurs in DT plants *in situ* remains unknown. Certainly the xylem tissue must remain intact for full hydraulic recovery of existing tissues to take place. Sherwin and Farrant (1996) suggest that xylem embolism may impede the recovery of photosynthetic organs in some DT plants. Since embolism recovery appears to be a metabolically expensive process (Zwieniecki and Holbrook, 2009; Brodersen et al., 2010), desiccation tolerant tracheophytes are typically short statured with xylem conduits that are probably much narrower than those of non-DT plants to facilitate conduit refilling and re-establish photosynthesis. Although embolism repair in DT and non-DT sporophytes remains unresolved, drought tolerance, whether through adaptive spatial organization of the xylem or an embolism repair mechanisms must have been a key feature of early-diverging tracheophytes.

## Desiccation tolerance in the gametophyte

An important aspect that separates ferns from seed plants is the presence of an independent haploid gametophyte stage. Unlike the gametophytes of angiosperms and gymnosperms, the fern gametophyte is a photosynthetic free-living entity that is often portrayed as a small, simple, delicate, ephemeral stage of the fern lifecycle. This stage may be considered anatomically simple with most taxa producing gametophytes that are a single cell layer thick, lack stomata, and vascular tissues, and produce a rudimentary cuticle (Watkins and Cardelús, 2012). Yet, a different picture emerges for overall gametophyte morphology. Most species produce gametophytes whose morphology differs significantly from the textbook cordiform thallus. Gametophyte form varies from filamentous as in some *Hymenophyllum*, to strap and ribbon-shaped (type II and III, respectively, *sensu* Farrar et al., 2008) in some Polypodiaceae and Vittariaceae. These non-cordiform types produce large, complex, three-dimensional gametophytes that are often perennial and can persist for decades as an independent organism (Watkins et al., 2007a,b; Watkins and Cardelús, 2012). As every sporophyte owes its existence to the one or more gametophytes that preceded it in that habitat, a discussion of fern biology would be incomplete without consideration of the gametophyte stage. Indeed, gametophyte form, physiology, and species reproductive biology may be intrinsically linked with sporophyte distribution and the species' niche.

Contrary to nonsensical textbook depictions of the selfing fern gametophyte that equates dispersal with migration, studies have shown that most sporophytes are generated through outcrossing pathways (e.g., Chiou et al., 1998, 2002; Yatabe et al., 2002). A “selfed” sporophyte would be 100% homozygous at all loci and most individuals maintain sufficient genetic load to prohibit such mating [although notable exceptions exist: e.g., *Asplenium platyneuron* (Crist and Farrar, 1983)]. Fern gametophytes are left with a fairly intractable situation of exchanging miniscule gametes across relatively vast areas. Dassler and Farrar (1997) and Farrar (1998) have argued that indeterminate branching thalli can form gametophyte banks that “wait” for compatible gamete arrival. This may be accomplished through additional spore migrants, or from two gametophytes growing into contact with each other. Perennial gametophytes can obtain sizes of several square centimeters and produce dozens of sporophytes over space and time (Farrar et al., 2008; Watkins and Cardelús, 2012). Given their relative simplicity, long-lived gametophytes face a unique set of physiological challenges relative to sporophytes.

**Figure 4:**
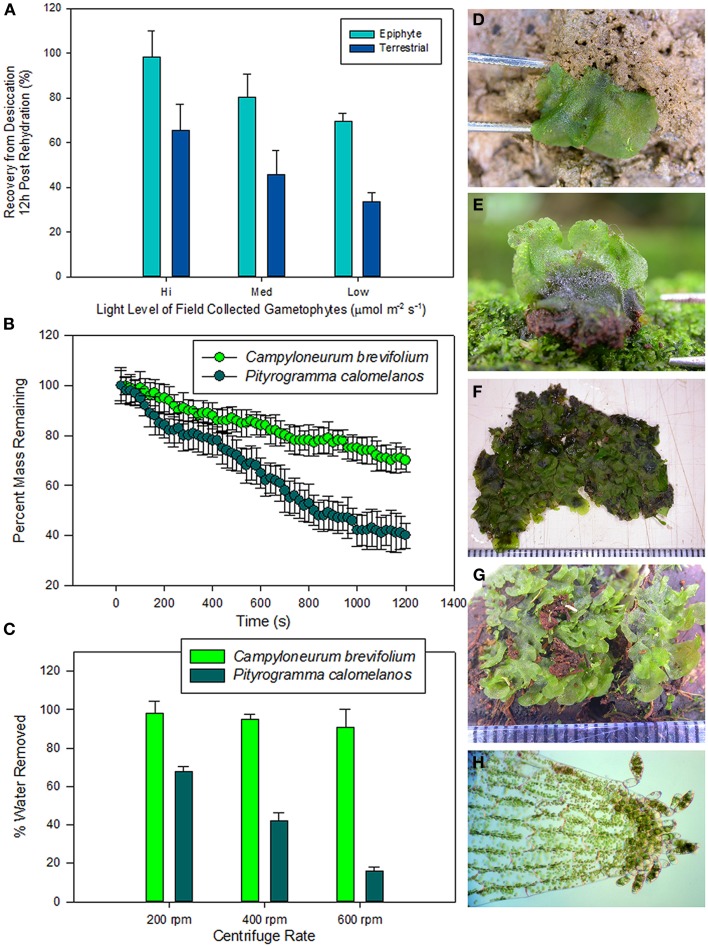
**(A)** Desiccation recovery survey of 20 species of field-collected fern gametophyes from the La Selva Biological Station in Costa Rica. Species were dried to equilibrium at −94 MPa and remained in that state for 96 h. Gametophytes were then rehydrated and measurements of chlorophyll fluorescence (*F*_*v*_/*F*_*m*_) were taken 12 h post rehydration. **(B)** Gametophyte drying curves of two fern species: *Campyloneurum brevifolium* a high light epiphyte that produces three-dimensional and overlapping gametophytes typical of epiphytic species and *Pityrogramma calomelanos* a high light terrestrial species that produces cordiform gametophytes typical of terrestrial species. Curves were created by adding 0.2 ml of water to ten field collected gametophytes of similar initial mass and the thalli were allowed to dry at −45MPa for 20 min. **(C)** Exohydric water holding capacity of gametophytes generated by spinning gametophytes in microcentrifuge spin columns. **(B)** above. Field collected gametophytes of: **(D)** *Pityrogramma calomelanos* (terrestrial high light), **(E)** *Cyclopeltis semicordata* (terrestrial low light), **(F)** *Campyloneurum brevifolium* (epiphytic low light), **(G)** *Elaphoglossum c.f. latifolium* (epiphytic high light), **(H)** *Radiovittaria stipitata* (epiphytic high light with asexual gemmae).

With limited internal water capacitance and poorly developed cuticle, fern gametophytes are in a constant state of equilibrium with their surrounding microenvironment (Watkins et al., 2007a,b). In response to these challenges, some gametophytes have developed remarkable degrees of desiccation tolerance. Such tolerance is exceedingly rare in fern sporophytes with less than 1% exhibiting this phenomenon. Yet, many free-living fern gametophytes exhibit extreme degrees of DT. In a survey of 20 field-collected species from the La Selva Biological Station in Costa Rica, it was found that, following rehydration from a 96 h dry down period at −94 MPa, all species exhibited some degree of physiological recovery (Figure 4). Whereas few environmental parameters were measured, the ability of a species to recover was linked to both life-form, epiphytes had greater recovery than terrestrial species, and to a species' light environment, taxa from higher light habitats exhibited greater recovery in general. Similar patterns have been found with more dramatic recovery from lower water potentials. For example, Watkins et al. (2007a,b) showed that the gametophytes of the twig epiphyte *Microgramma percussa* exhibited complete recovery after 12-h rehydration following drying to −219 MPa. Far from being stress sensitive entities, many fern gametophytes cope well with water stress.

In addition to the physiological adaptations related to DT, fern gametophyte morphology is closely linked to habitat and may be linked to ecophysiology. In a study on tropical fern gametophytes, Watkins et al. (2007a,b) showed that desiccation recovery was closely linked to the rate at which gametophytes dried. When subjected to identical conditions, increased tolerance was observed in species that dried more slowly. With no cuticle or stomata, they argued that changes in boundary layer and exohydric water holding capacity driven by gametophyte morphology could be an effective mechanism influencing drying rate (see also Watkins and Cardelús, 2012). Complex thalli with three-dimensional shapes likely have deeper boundary layers and smaller angles that hold onto water to slow water loss. For example, 0.2 ml of water was added to field collected gametophytes of similar initial mass and the thalli were allowed to dry at −45 MPa for 20 min. The gametophytes of *Campyloneurum brevifolium*, a canopy epiphyte with complex three dimensional morphology dried at a much slower rate than did *Pityrogramma calomelanos*, a terrestrial species with planar cordate morphology (Figure 4). When centrifuged in spin columns, *C. brevifolium* held onto a greater percentage of externally stored water than did *P. calomelanos*. A similar response was found for other species with similar morphologies. It appears that gametophyte morphology may play an important role in water relations. Interestingly, taxa that produce morphologically complex thalli are frequently limited to xeric epiphytic and some xeric terrestrial habitats (Watkins and Cardelús, 2012). The physiological ramifications of external water holding on fern gametophytes have yet to be examined.

The independent gametophyte generation is a critical exploratory stage for ferns. Gametophytes, in most all cases, are more stress tolerant than sporophytes and can grow in areas where sporophytes cannot. In much the same way as seed banks, gametophyte banks can wait out stressful environmental periods or periods when proper genetic resources are not available for recruitment. Interestingly, adaptations in gametophyte morphology and physiology that provide for extreme stress tolerance and indeterminate growth are particularly well-developed in epiphytic taxa. The general instability of the terrestrial habitat (from erosion, leaf litter, animal disturbance etc.) may have prevented the evolution of large long-lived gametophyte in terrestrial species. Critically needed are additional studies on terrestrial species' ecology.

## Epiphytism and radiation into diverse niches

Based on the current understanding of Pteridophyte evolution, leptosporangiate ferns experienced dramatic species diversification during the late Mesozoic into the Cenozoic (Schneider et al., 2004; Schuettpelz and Pryer, 2009). Not only did this period see an increase in species richness, but also the widespread appearance of an array of taxonomically diverse epiphytic flora. There are a number of proposed mechanisms behind this radiation into the forest canopy including climate change and competition for light from angiosperms (reviewed in Zachos et al., 2008; Watkins and Cardelús, 2012) yet, regardless of how this happened, it is important to understand the novel challenges that ferns would have faced as they radiated into epiphytic conditions. The epiphytic habitat lies in stark contrast to the forest floor since epiphytic habitats are characterized by high variation in microclimate, with generally higher light levels, lower humidity, and higher temperatures than the forest floor (Cardelús and Chazdon, 2005; Watkins and Cardelús, 2009). Yet ferns (along with many angiosperms) appear to have radiated into these habitats in great numbers. In the case of ferns, this is all the more intriguing given the functional adaptations that had to occur in both the independent gametophyte and sporophyte.

Using our modern flora as a model, ferns would have needed to evolve novel ecophysiological traits in both the gametophyte and sporophyte generations to be successful in the epiphytic niche. Importantly, the physiological co-ordination of the sporophyte and gametophyte stages must have supported the radiation of ferns into the canopy. Epiphytic ferns produce gametophytes that are markedly more desiccation tolerant, less sensitive to high light stress, and potentially use different sources of nitrogen than terrestrial species (Farrar et al., 2008; Watkins and Cardelús, 2012). Consistent with their habitat, epiphytic sporophytes have significantly more resistive vascular systems, greater protection from cavitation, increased drought tolerance, and fundamentally different morphologies (among several other differences) relative to terrestrial species (Hietz and Briones, 1998; Watkins et al., 2010; Watkins and Cardelús, 2012). Similar traits may have been acquired in epiphytic angiosperms, yet the dual ecological life history of ferns posed a novel set of constraints that would not have been required in seed plants.

Similar to fern epiphytes, we suspect that evolution selected for adaptive traits in both sporophytes and gametophytes found in habitats outside of the mesic niche. For example, the Pteridaceae family of ferns exploits epiphytic, terrestrial, xeric, alpine and aquatic niches that challenge standard ideas of fern biogeography (Schuettpelz et al., 2007; Hietz, 2010) and invite deeper inquiry into the physiological tolerances of pteridophytes in general. Within the Pteridaceae, members of the Cheilanthoid clade occupy the seasonally wet deserts of the American south-west, and exhibit traits such as hirsute fronds and scaly cuticles to increase albedo and ultimately reduce water loss, while other species bear abaxial trichomes to facilitate foliar water absorption. Do Cheilanthoids tolerate or evade drought, or is there a continuum of water relations strategies? What can we expect of Cheilanthoid gametophytes? By contrast to these desert taxa, the Ceratopteridoid clade is restricted to freshwater swamps and mangroves, while the Vittarioids are exclusively epiphytic, with some species exhibiting CAM photosynthesis (Martin et al., 2005; Schuettpelz et al., 2007; Hietz, 2010). Are members of the Pteridaceae physiologically or structurally pre-disposed to tolerate water deficit? The reasons behind the adaptive radiation of the Pteridaceae ferns remain unclear, but trait mapping and ancestral reconstructions using a fossil-calibrated phylogeny may provide useful clues for elucidating the mechanisms behind the diversification of Pteridaceous ferns.

## Conclusion and future directions

There is good reason to suspect that physiological and morphological traits in both the sporophyte and gametophyte stages in a coordinated manner that was consistent with the Cenozoic diversification of the fern epiphytic flora, and possibly other fern radiations across the post-Eocene landscape. Future research will seek to elucidate the structure-function trade-offs associated with variable stele structure and pit membrane traits with respect to hydraulic function and cavitation resistance, since so little is known about the functional value of the myriad of bundle arrangements characteristic of fern taxa. Since the fern vascular system is anatomically tractable, future work will explore the relationships between stele arrangements, frond venation and gas-exchange, with the goal of placing the results in a broad phylogenetic context. Working toward a concurrent understanding of how gametophyte physiology parallels adaptive traits in the sporophyte will be critical toward building a comprehensive picture of fern radiations since gametophyte establishment may push the physiological and niche boundaries of their associated sporophytes (Watkins et al., 2007a,b; Farrar et al., 2008).

Owing to their seedless, largely treeless habit and a lifestyle seemingly resigned to the shady forest understory, pteridophyte physiology has long lingered in the shadow of conifers and angiosperms. However, emerging work has shown ferns to physiologically competitive, resistant to stress, highly diverse and extremely adaptive, so it is time for this ancestral lineage to step into the spotlight of evolutionary eco-physiology. Indeed, our understanding of the evolution of plant water transport would be incomplete without a vigorous examination of the physiological traits that contributed to the 400 million year-long success of these persistent plants.

### Conflict of interest statement

The authors declare that the research was conducted in the absence of any commercial or financial relationships that could be construed as a potential conflict of interest.
